# 19% Efficient P3CT-Na Based MAPbI_3_ Solar Cells with a Simple Double-Filtering Process

**DOI:** 10.3390/polym13060886

**Published:** 2021-03-13

**Authors:** Shou-En Chiang, Qi-Bin Ke, Anjali Chandel, Hsin-Ming Cheng, Yung-Sheng Yen, Ji-Lin Shen, Sheng Hsiung Chang

**Affiliations:** 1 Department of Physics, Chung Yuan Christian University, Taoyuan 32023, Taiwan; 10762004@cycu.org.tw (S.-E.C.); abcd311128@gmail.com (Q.-B.K.); anjalichandel62@gmail.com (A.C.); jlshen@cycu.edu.tw (J.-L.S.); 2 Department of Electronic Engineering and Organic Electronics Research Center, Ming Chi University of Technology, Taipei 24301, Taiwan; 3 Department of Chemistry, Chung Yuan Christian University, Taoyuan 32023, Taiwan; ysyen@cycu.edu.tw; 4 R&D Center for Membrane Technology and Center for Nanotechnology, Department of Physics, Chung Yuan Christian University, Taoyuan 32023, Taiwan

**Keywords:** P3CT-Na thin film, zetapotentials, MAPbI_3_ solar cells, double-filtering process, interfacial contact

## Abstract

A high-efficiency inverted-type CH_3_NH_3_PbI_3_ (MAPbI_3_) solar cell was fabricated by using a ultrathin poly[3-(4-carboxybutyl)thiophene-2,5-diyl]-Na (P3CT-Na) film as the hole transport layer. The averaged power conversion efficiency (PCE) can be largely increased from 11.72 to 18.92% with a double-filtering process of the P3CT-Na solution mainly due to the increase in short-circuit current density (*J_SC_*) from 19.43 to 23.88 mA/cm^2^, which means that the molecular packing structure of P3CT-Na thin film can influence the formation of the MAPbI_3_ thin film and the contact quality at the MAPbI_3_/P3CT-Na interface. Zeta potentials, atomic-force microscopic images, absorbance spectra, photoluminescence spectra, X-ray diffraction patterns, and Raman scattering spectra are used to understand the improvement in the *J_SC_*. Besides, the light intensity-dependent and wavelength-dependent photovoltaic performance of the MAPbI_3_ solar cells shows that the P3CT-Na thin film is not only used as the hole transport layer but also plays an important role during the formation of a high-quality MAPbI_3_ thin film. It is noted that the PCE values of the best P3CT-Na based MAPbI_3_ solar cell are higher than 30% in the yellow-to-near infrared wavelength range under low light intensities. On the other hand, it is predicted that the double-filtering method can be readily used to increase the PCE of polymer based solar cells.

## 1. Introduction

The photovoltaic performance of perovskite solar cells has satisfied the requirements for commercialization, which can provide the power conversion efficiencies (PCE) of 25.5% [1] and 22.3% [2] when the regular-type and inverted-type device architectures are used, respectively. In regular-type perovskite solar cells, TiO_2_ and SnO_2_ thin films are widely used as the electron transport layer (ETL) to collect (block) the photo-generated electrons (holes) from the perovskite thin film. The formation of high-quality n-type inorganic materials always requires a high-temperature annealing process. For example, the sintering temperature of anatase TiO_2_ is about 450 °C [3], which can damage plastic substrates and thereby impeding the application in flexible optoelectronic devices. In inverted-type perovskite solar cells, poly(3,4-ethylenedioxythiophene):polystyrenesolfonate (PEDOT:PSS) thin films are widely used as the hole transport layer (HTL) to collect (block) the photo-generated holes (electrons) from the perovskite thin film. Fortunately, PEDOT:PSS thin films can be fabricated by using various solution process methods under low temperatures (<120 °C). Besides, it is naturally to form a smooth contact between the hydrophilic PEDOT:PSS and perovskite thin films, which results in a high-efficiency hole collection and thereby contributing to the generation of photocurrent [4]. However, the Fermi level of PEDOT:PSS thin films is ranging from 5 to 5.1 eV [5,6,7], which results in a relatively low open-circuit voltage (V_OC_) from 0.85 to 0.95 V [8,9,10]. Wide-bandgap p-type NiO_x_ and CuO_x_ thin films were used to replace PEDOT:PSS thin films as the HTL of inverted-type perovskite solar cells, which results in the higher V_OC_. However, the short-circuit current density (*J_SC_*) of the resultant perovskite solar cells is lower than 22.5 mA/cm^2^ [11,12,13,14] due to the lower hole-collection efficiency from the perovskite thin film to the inorganic HTL. In recent years, the poly [3-(4-carboxybutyl)thiophene-2,5-diyl] (P3CT) based thin films are also used as the efficient HTL in inverted-type perovskite solar cells [15,16,17,18]. The P3CT based polymers have hydrophilic side chains, which can be partially dissolved in water solutions. Therefore, the spin-coating method can be used to fabricate P3CT based thin films on top of the ITO/glass or ITO/PET substrate with a post thermal annealing process under low temperatures. The use of an ultra-thin P3CT-X (X: Na^+^, K^+^, Rb^+^, Cs^+^, or CH_3_NH_3_^+^) film can be considered as the modification layer on top of the conductive ITO, which modifies the work function of the transparent electrode to efficiently collect the photo-generated holes from the perovskite thin film. It is noted that the V_OC_ and *J_SC_* of the P3CT-X based perovskite solar cells can be higher than 1.05 V and 24 mA/cm^2^, respectively. However, the formation of large-scale and ultrathin P3CT-X films was not investigated in literatures [15,16,17,18]. It gives the reason to investigate the relation between the morphology of the P3CT-X thin films and the photovoltaic performance of the resultant perovskite solar cells. Our main goal is to form a large-scale and ultrathin P3CT-X interlayer for the realization of high-efficiency inverted-type perovskite solar cells. The experimental results show that the high-efficiency perovskite solar cells rely on the use of a double-filtering method for the P3CT-Na solution. A high PCE of 19.03% was achieved in the inverted-type perovskite solar cells when the double-filtering process of P3CT-Na solution is used to modify the molecular packing structure of the HTL. On the other hand, it is predicted that the double-filtering method can be readily used to increase the PCE of polymer based solar cells [19,20].

## 2. Experiments

P3CT-Na/water solution was prepared through the chemical reaction of poly[3 -(4-carboxybutyl)thiophene-2,5-diyl] (P3CT) (regioregularity of 86%, Rieke, Auburn, INdia) with dry NaOH (99.99%, Alfa Aesar, Haverhill, MA, USA). 9 mg P3CT and 1.947 mg NaOH were added in to a 4.5 mL deionized (DI) water and a 0.987 mL DI water, respectively. To increase the dissolving ability of P3CT polymers in water, the solution was treated by ultrasonic treatment for 30 min. The NaOH/water solution was slowly added into the dark purple P3CT/water solution in order to minimize the thermal effects from the exothermic interaction. Then, the mixture of P3CT/water and NaOH/water was stirred at 40 °C for 6 h. After the chemical interaction, the estimated concentration of P3CT-Na/water is about 1.54 mg/mL. CH_3_NH_3_PbI_3_ (MAPbI_3_) precursors were prepared by dissolving MAI transparent crystals (0.2384 mg) and PbI_2_ brown powders (0.6915 mg) in a 1-mL Dimethylformmamide (DMF)/dimethyl sulfoxide (DMSO) mixture (9:1, *v*/*v*), which resulted in a 1.5 M MAPbI_3_ precursor solution. A total of 10 mg (6,6)-PhenylC61butyricacidmethylester (PCBM) powder (99.5%, Uni-Onward, Taipei City, Taiwan) was dissolved in a 0.5-mL chlorobenzene (CB). 0.01-mg bathocuproine (BCP) power (99%, Seedchem, Camberwell, Austalia) was dissolved in a 0.5 mL isopropanol (IPA) (99.5%, ACROS, Fisher Scientific, Waltham, MA, USA). The MAPbI_3_ precursor solution, PCBM/CB solution (2 wt.%) and BCP/IPA (0.05 wt.%) solutions were stirred at 400 rpm for 4 h under room temperatures.

**Figure 1:**
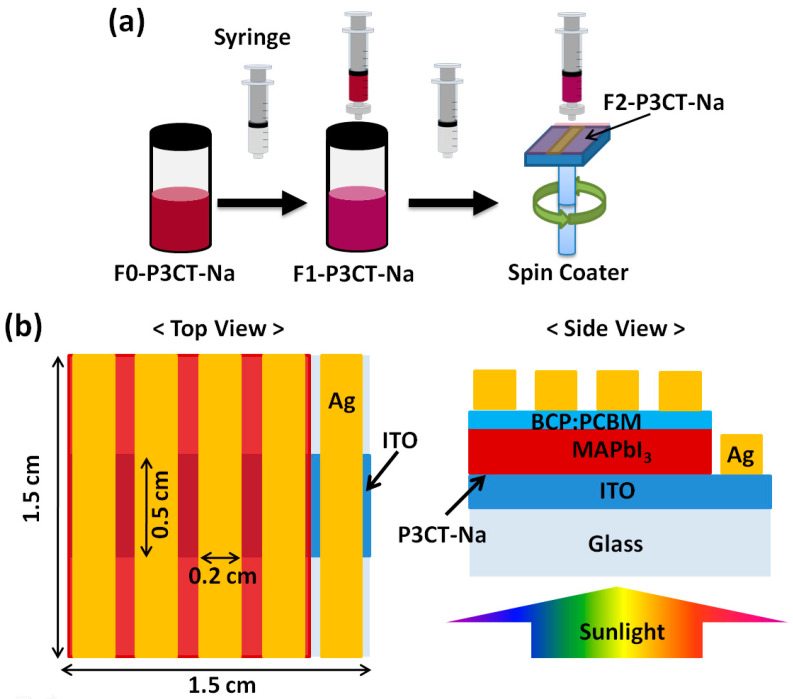
(**a**) Single-filtering and double-filtering processes for the P3CT-Na water solution. (**b**) Top view and side view of the device structure.

In the device fabrication, the ITO/glass (7 Ω/sq) samples were treated by using a UV-Ozone cleaner for 50 min. Before the deposition of the P3CT-Na thin films, the P3CT-Na solutions were treated with and without the filtering processes, as shown in Figure 1a. The averaged pore diameter of the used filters is 0.45 μm. Without the filtering process, it is denoted as F0-P3CT-Na solution. With the single-filtering (double-filtering) process, it is denoted as F1-P3CT-Na (F2-P3CT-Na) solution. Additionally, the ITO/glass samples were heated at 120 °C for 10 min in order to remove the residual moisture on the substrates. Then, the different P3CT-Na solutions were spin-coated on top of the ITO/glass samples with a post thermal annealing at 140 °C for 20 min, which can be used as the HTL. The spin rate and time of the P3CT-Na solutions are 4000 rpm and 60 s, respectively. The thickness of P3CT-Na thin films is about 4 nm [15]. The surface area of the ITO/glass samples is 2.25 cm^2^. The 600-nm thick MAPbI_3_ thin films were fabricated on top of the P3CT-Na/ITO/glass sample by using the one-step spin coating method with a washing-enhanced nucleation process (WEN) [21,22,23]. In the WEN process, the dropping time and anti-solvent are the last 20 s and CB, respectively. The 50 nm thick closely-packed PCBM small molecules thin films were spin-coated on top of the MAPbI_3_/P3CT-Na/ITO/glass samples to act as the ETL. The BCP/IPA solutions were spin-coated on top of the PCBM/MAPbI_3_/P3CT-Na/ITO/glass samples as an electron modification layer (or electron buffer layer) in order to increase (decrease) the electron-collection efficiency (carrier recombination) at the PCBM/MAPbI_3_ interface [24]. The spin speeds of the PCBM/CB and BCP/IPA solutions on top of the samples are 1250 and 4000 rpm, respectively. Then, the 100 nm thick Ag thin films were thermally deposited on top of the BCP:PCBM/MAPbI_3_/PEDOT:PSS/ITO/glass samples with low deposition rates (<0.1 nm/s). The active area of the P3CT-Na based MAPbI_3_ solar cells is defined to be 0.2 cm × 0.5 cm with the use of a metallic shadow mask during the thermal evaporation process of Ag thin film. One sample contains four cells, as shown in Figure 1b.

In the device characterization, The J-V curves of the solar cells were measured by using a source-meter system (NI-USB 6356 DAQ, National Instruments, Austin, TE, USA) under one sun illumination (AM1.5G, 100 mW/cm^2^). The intensity-dependent and wavelength-dependent photovoltaic responses of the solar cells were measured by using a source-meter system (NI-USB 6356 DAQ, National Instruments, Austin, TE, USA). The light intensity of the light-emitting diodes based solar simulator (VeraSol-2, Newport, OR, USA) was calibrated by using a reference cell (91150V, Newport, OR, USA).

In the materials characterizations, the different P3CT-Na solutions were characterized by using Zeta potential analyzer (ELSZ-2000, Otsuka, Tokyo, Japan). The P3CT-Na/ITO/glass samples were characterized by using a home-made transmittance spectrometer [9] and a commercial atomic-force microscope (Innova, Bruker, Billerica, MA, USA). The MAPbI_3_/P3CT-Na/ITO/glass samples were characterized by using a home-made transmittance spectrometer [9], a home-made photoluminescence spectrometer [24], a commercial X-ray diffractometer (D2 Phaser, Bruker, Billerica, MA, USA) and a commercial Raman scattering spectrometer (iHR550, Horiba, Kyoto, Japan) with a 532 nm excitation laser.

## 3. Results and Discussion

**Figure 2:**
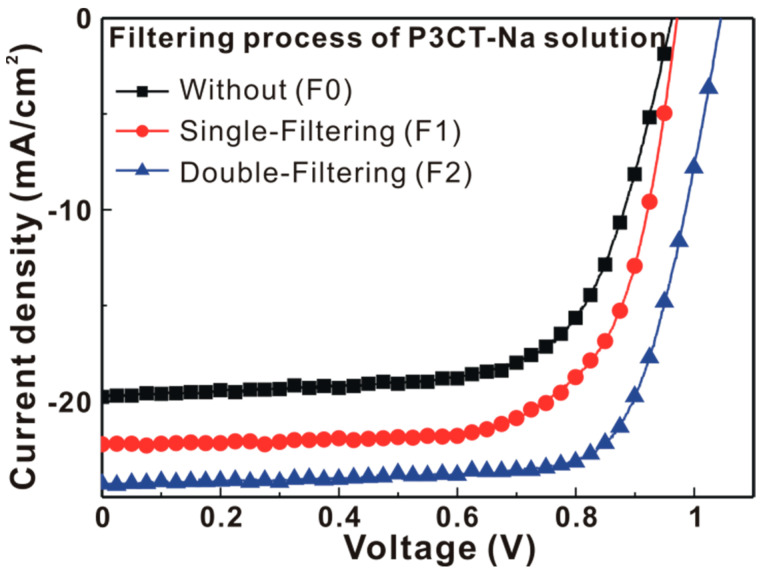
J-V curves of the P3CT-Na based MAPbI_3_ solar cells with the different filtering processes under one sun illumination (AM 1.5G, 100 mW/cm^2^).

Figure 2 presents the current density-voltage (J-V) curves of the P3CT-Na based MAPbI_3_ solar cells with the different P3CT-Na solutions. The averaged photovoltaic performance of 16 cells for each condition is listed in Table 1. Without the use of filtering process for the P3CT-Na solution, the photovoltaic performance of the resultant solar cells has the largest deviation in the *J_SC_*, which results in a moderate PCE of 11.83%. When the F1-P3CT-Na solution was used to prepare the P3CT-Na thin film, the averaged PCE significantly increased from 11.72 to 14.63% mainly due to the increase in the averaged *J_SC_* from 19.43 to 22.16 mA/cm^2^. When the F2-P3CT-Na solution was used to prepare the P3CT-Na thin film, the averaged PCE largely increased to 18.92% due to the increases in the *J_SC_* and fill factor (FF). It is noted that the standard deviation in the *J_SC_* significantly decreases from 2.32 to 0.17 mA/cm^2^, which means that a large-scale and ultrathin P3CT-Na film can be formed when the double-filtering process is used to reduce the aggregation of P3CT-Na polymers. Besides, the trend of the Voc values is proportional to the trend of the FF, which indicates that the potential loss is mainly from the non-radiatively recombination in the MAPbI_3_ thin film and at the MAPbI_3_/P3CT-Na interface. The detailed device characterizations can be found in Figure 9.

**Figure 3:**
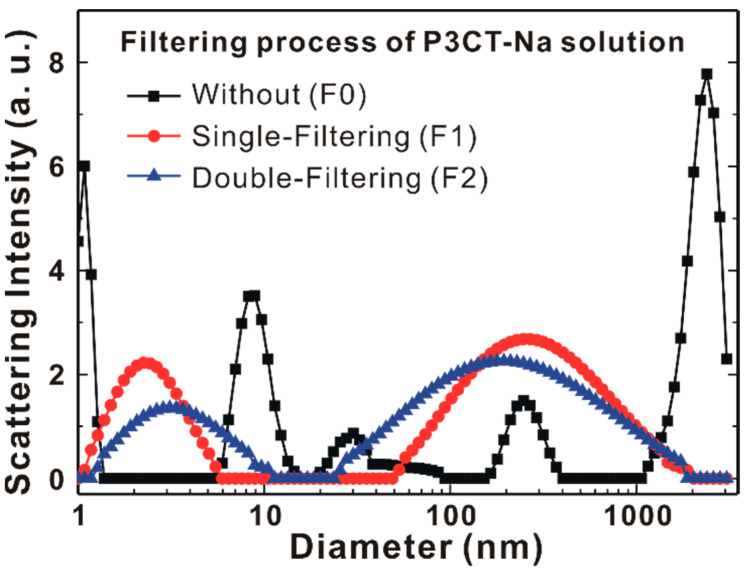
Particle sizes of aggregated P3CT-Na polymers in water solutions without and without the filtering processes.

Figure 3 presents the particle diameter dependent electrophoretic light scattering intensity of the different P3CT-Na water solutions. Without the filtering process, the particle diameters of the aggregated P3CT polymers have the most diverse distributions from 1 nm to 2.4 μm, which means that the resultant P3CT thin film on top of the ITO/glass substrate can contain micrometer-sized P3CT-Na aggregates. When the single-filtering process (F1) is used to remove the micrometer-sized P3CT-Na aggregates with a hydrophilic polyvinyl difluoride (PVDF) syringe filter (0.45 μm pore), the diverse particle diameters of the aggregated P3CT polymers are reduced to 2.3 and 254 nm. It can be understood that the micrometer-sized P3CT-Na aggregates (isolated P3CT-Na polymers) are broken up (packed together) after the single-filtering process. After the double-filtering process (F2), the distributions of the nanometer-sized P3CT-Na aggregates and sub-micrometer-sized P3CT-Na aggregates both become broader. It is noted that the diameter of the nanometer-sized P3CT-Na aggregates increased from 2.3 to 3.5 nm, which is close to the optimal thickness of the P3CT-Na thin film as the HTL of perovskite solar cells [15].

**Figure 4:**
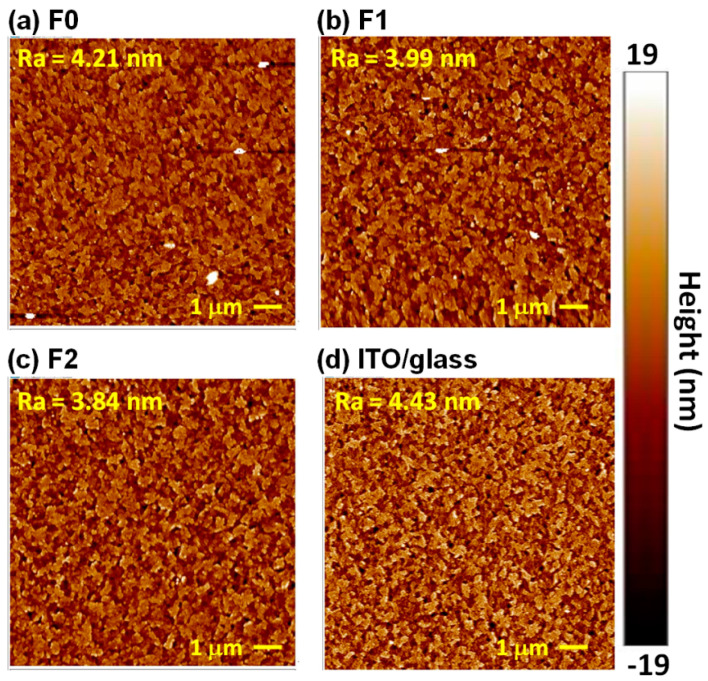
AFM images of P3CT-Na/ITO/glass substrates. (**a**) Without filter; (**b**) With single-filtering process; (**c**) With double-filtering process. (**d**) ITO/glass substrate.

Figure 4 presents the AFM images of the P3CT-Na/ITO/glass and ITO/glass samples. The surface roughness (Ra) of the ITO/glass sample is 4.43 nm. Without the filtering process, the surface obviously contains micrometer-sized particles (see Figure 4a), which can be explained as due to the existence of micrometer-sized P3CT-Na aggregates in the precursor solution (see Figure 3). When the single-filtering process is used, the surface only contains a few micrometer-sized particles (see Figure 4b). There are almost no particles in the surface of the P3CT-Na/ITO/glass sample (see Figure 4c) when the double-filtering process is used, which means that the sub-micrometer-sized P3CT-Na aggregates are not deposited on top of the UV-Ozone treated hydrophilic ITO surface [25] and thereby suggesting that the sub-micrometer-sized P3CT-Na aggregates are hydrophobic. Besides, the Ra of the P3CT-Na/ITO/glass samples decreases from 4.21 to 3.84 nm by increasing the number of filtering process, which means that the coverage of P3CT-Na thin film on top of the sub-microstructured ITO film can be improved by reducing the micrometer-sized P3CT-Na aggregates. In other words, the hydrophilic UV-Ozone treated ITO surface is modified by the nanometer-sized P3CT-Na aggregates thin film, which also shows that broader distribution of the nanometer-sized P3CT-Na aggregates (see Figure 3) is more suitable for covering the sub-microstructured ITO thin film when the double-filtering process is used.

**Figure 5:**
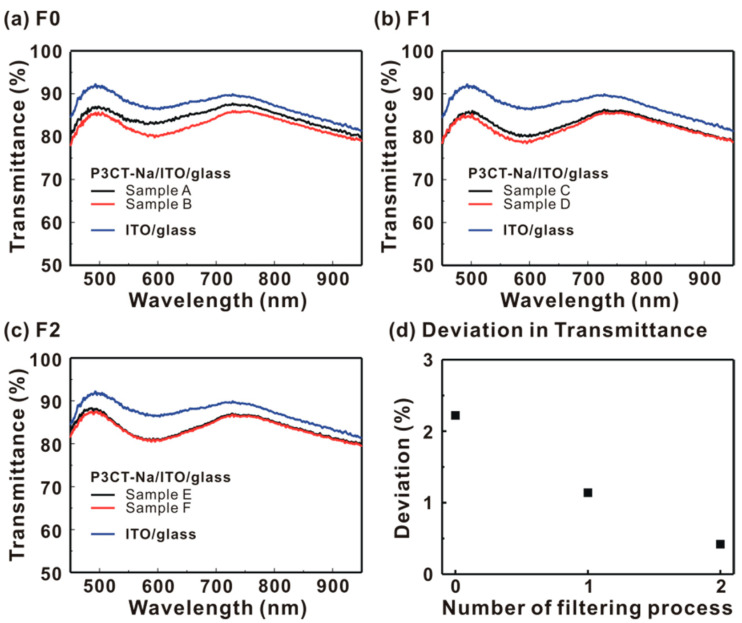
Transmittance spectra of P3CT-Na/ITO/glass substrates. (**a**) Without filter; (**b**) With single-filtering process; (**c**) With double-filtering process. (**d**) Averaged deviation in transmittance spectra from 450 to 650 nm.

Figure 5 presents the transmittance spectra of the P3CT-Na/ITO/glass and ITO/glass samples. When the P3CT-Na thin films are deposited on top of the ITO/glass samples, the transmittance values in the visible wavelength range and in the near-infrared wavelength range are both decreased mainly due to the light scattering and absorption from the P3CT-Na thin films, respectively. Without the filtering process, the largest deviation in the transmittance spectra of the two P3CT-Na/ITO/glass samples (samples A and B) is 3% at the wavelength of 597 nm due to the different light absorption from the P3CT-Na thin film, which indicates that the F0-P3CT-Na water solution is not uniform. When the single-filtering process is used, the largest deviation in the transmittance spectra of the P3CT-Na/ITO/glass samples (samples C and D) significantly decreased to 1.3%. The transmittance spectra of the two P3CT-Na/ITO/glass samples (samples E and F) are almost overlapped, which indicates that the nanometer-sized P3CT-Na aggregates are uniformly suspended in the water solution and thereby resulting in the highly repeatable P3CT-Na thin films on top of the ITO/glass samples. The overlapped transmittance spectra of samples E and F also can be used to explain the relatively low deviation in the *J_SC_* (see Table 1).

**Figure 6:**
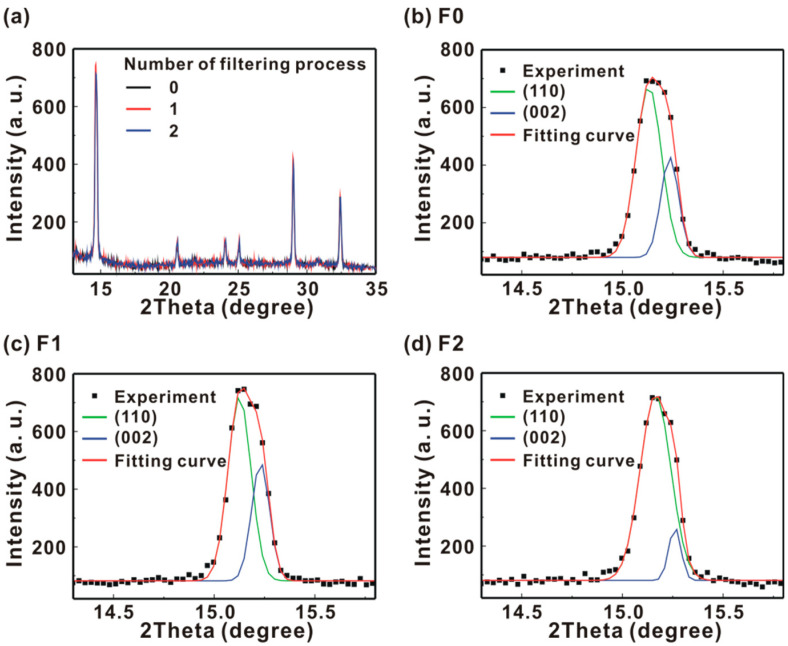
(**a**) X-ray diffraction patterns of the MAPbI_3_/P3CT-Na/ITO/glass samples. Main diffraction peaks of the MAPbI_3_ thin films at (110) and (002): (**b**) without filtering process; (**c**) single-filtering process; (**d**) double-filtering process.

**Figure 7:**
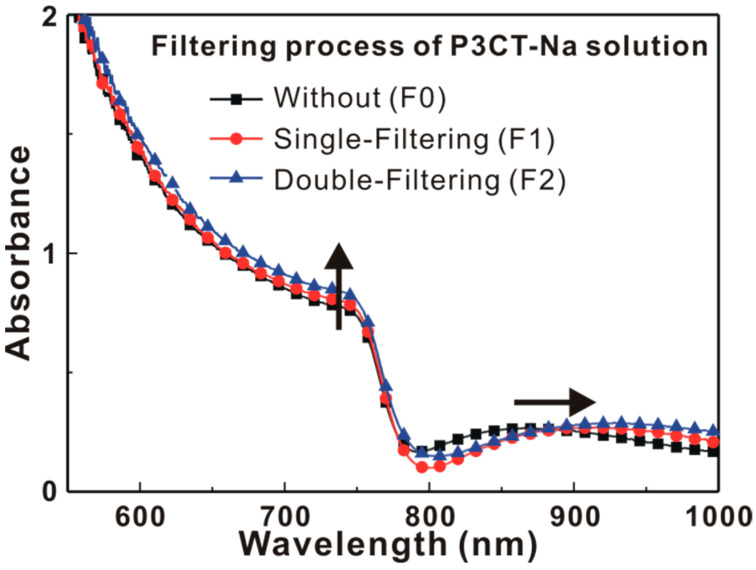
Absorbance spectra of the MAPbI_3_/P3CT-Na/ITO/glass samples.

**Figure 8:**
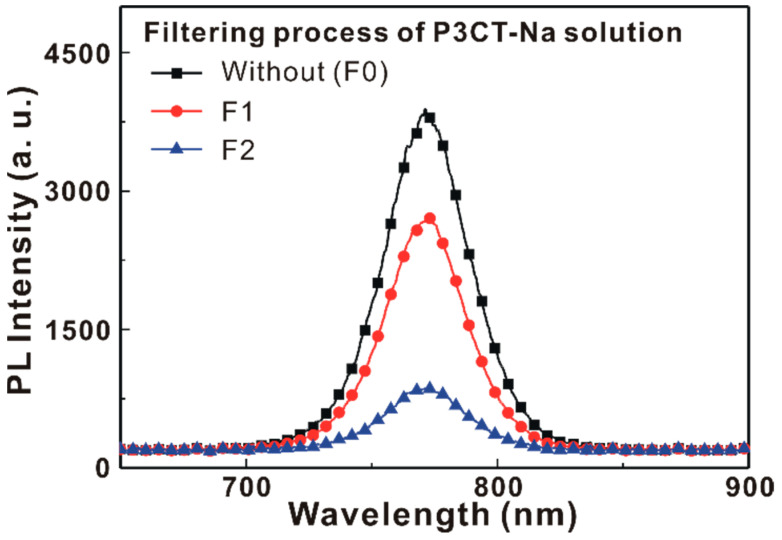
Photoluminescence spectra of the MAPbI_3_/P3CT-Na/ITO/glass samples.

Figure 6 presents the X-ray diffraction patterns of the MAPbI_3_/P3CT-Na/ITO/glass samples. Figure 6a shows that the six diffraction peaks can be used to confirm the formation of tetragonal perovskite crystal structure [26]. The intensities of the main diffraction peak are almost independent the properties of the P3CT-Na thin films, which means that the crystallinities of these MAPbI_3_ thin films are similar and thereby resulting in the relatively small deviations in the V_OC_ and FF of the resultant solar cells (see Table 1). The main diffraction feature of the MAPbI_3_ thin films is fitted with a dual Gaussian equation, as shown in Figure 6b,c, which can be assigned to the diffraction peaks at (110) and (220). When the F2-P3CT-Na solution is used, the main diffraction peak of the resultant MAPbI_3_ thin film is (110), and the broader diffraction peak at (110) means the better contact at the interface between the MAPbI_3_ crystal and P3CT-Na polymer. Figure 7 presents the absorbance spectra of the MAPbI_3_/P3CT-Na/ITO/glass samples. The absorbance values in the light absorption band of the MAPbI_3_ thin films increase with the number of the filtering process for the P3CT-Na solution. Additionally, the interference peak wavelength in the transparent spectra of the MAPbI_3_ thin films increases with the number of the filtering process for the P3CT-Na solution. The changes in the absorbance spectra of the MAPbI_3_ thin films means that the thickness of the MAPbI_3_ thin film increases with the number of the filtering process for the P3CT-Na solution [27]. Figure 8 presents the photoluminescence (PL) spectra of the MAPbI_3_/P3CT-Na/ITO/glass samples. The intensity of the PL spectra decreases with the number of the filtering process for the P3CT-Na solution. At the wavelength of the green excitation laser (λ = 532 nm), the absorbance (optical density) values of the MAPbI_3_/P3CT-Na/ITO/glass samples are higher than 2 (see Figure 7). In other words, the excitation laser can be effectively absorbed to generate excitons in the MAPbI_3_ thin films. Therefore, the PL intensity from the MAPbI_3_/P3CT-Na/ITO/glass sample can be used to evaluate hole-collection ability from MAPbI_3_ to P3CT-Na. The lower PL intensity is due to the higher hole-collection efficiency at the MAPbI_3_/P3CT-Na interface. When the F2-P3CT-Na solution is used, the larger absorbance (see Figure 7) and the lower PL intensity (see Figure 8) can be used to explain the higher *J_SC_* (see Figure 2 and Table 1).

**Figure 9:**
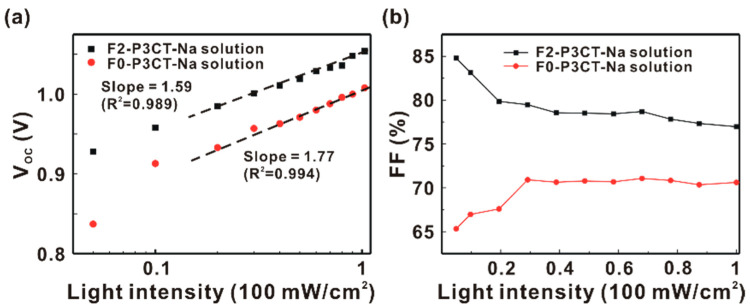
(**a**) Light-intensity dependent open-circuit voltage (V_OC_) of the P3CT-Na based MAPbI_3_ solar cells. (**b**) Light-intensity dependent fill factor (FF) of the P3CT-Na based MAPbI_3_ solar cells.

To explore the sunlight-generated carrier dynamics in the P3CT-Na based MAPbI_3_ solar cells, the light intensity-dependent V_OC_ and FF are measured from 5 to 100 mW/cm^2^, as shown in Figure 9. The slope (S) in Figure 9a can be used to evaluate the non-radiative recombination rate of the photo-excited carriers. The Koster equation [28,29,30] can be used to determine the slope:

(1) $$S=\frac{q}{K_{B}T}/(\frac{\partial V_{OC}}{\partial Ln(J_{SC})})$$

where *K_B_T* is the thermal energy, *q* is the electric charge and *J_SC_* is proportional to the sunlight intensity. The larger slope corresponds to the higher non-radiative carrier recombination rate in the solar cells. When the F2-P3CT-Na solution is used, the slope decreases from 1.77 to 1.59, which indicates that the non-radiative recombination of sunlight-generated carriers in the MAPbI_3_ thin film can be reduced by improving contact quality at the MAPbI_3_/P3CT-Na interface. The improved contact quality at the MAPbI_3_/P3CT-Na interface can be confirmed by the PL quenching experiments (see Figure 8) when the F2-P3CT-Na solution is used. Without the filtering process of the P3CT-Na solution, the FF gradually decreases with a decrease in the sunlight intensity from 30 to 5 mW/cm^2^ (see Figure 9b). When the sunlight intensity is higher than 30 mW/cm^2^, the FF remains a fixed value. It means that the defects of the solar cell fabricated without the F0-P3CT-Na solution can be filled completely by the photon-excited carriers when the sunlight intensity is higher than 30 mW/cm^2^. When the F2-P3CT-Na solution is used, there are two slopes in the light intensity-FF curve of the MAPbI_3_ solar cell. The FF slightly increases from 76.8 to 79.9% with a decrease in the sunlight intensity from 100 to 20 mW/cm^2^ due to the lower carrier recombination at the interfaces for thicker MAPbI_3_ layers [31]. It is noted that the FF significantly increases from 79.9 to 84.7% with a decrease in the sunlight intensity from 20 to 5 mW/cm^2^, which can be explained due to the MA dipoles-mediated carrier transportation behavior [32]. In other words, the electron and hole transportations are not influenced by the defects in the MAPbI_3_ thin film deposited on top of the P3CT-Na thin film coated ITO/glass sample when the F2-P3CT-Na solution is used.

**Figure 10:**
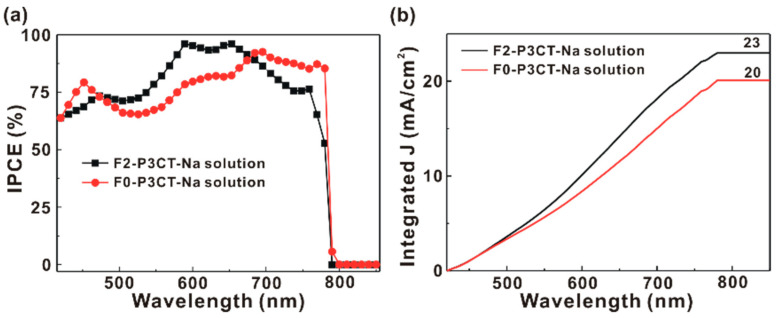
(**a**) Incident photon-to-current conversion efficiency spectra of the P3CT-Na based MAPbI_3_ solar cells. (**b**) Integrated photocurrent density (J) of the P3CT-Na based MAPbI_3_ solar cells.

**Figure 11:**
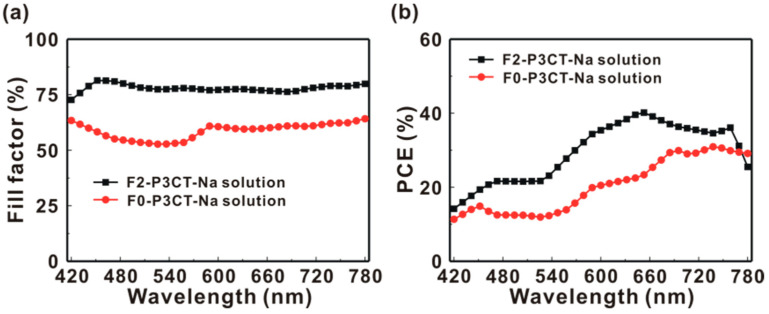
(**a**) Wavelength-dependent fill factor and (**b**) wavelength-dependent power conversion efficiency (PCE) of the P3CT-Na based MAPbI_3_ solar cells under a low light intensity of 4 mW.

**Figure 12:**
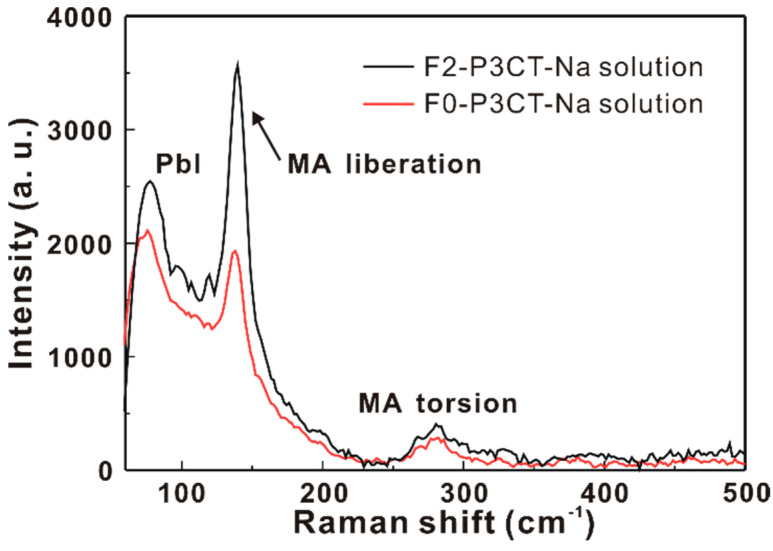
Raman scattering spectra from the MAPbI_3_/P3CT-Na interface under a green laser excitation with an objective lens (50×).

To investigate the wavelength-dependent photovoltaic performance, the J-V curves of the P3CT-Na based MAPbI_3_ solar cells are measured under the different excitation wavelengths from 420 to 850 nm at a fixed light intensity of 4 mW. Figure 10a presents the incident photon-to-current conversion efficiency (IPCE) spectra of the P3CT-Na based MAPbI_3_ solar cells fabricated with and without the double-filtering process for the P3CT-Na solution. The IPCE values are calculated from the measured *J_SC_* with the simple relation:

(2) $$\mathrm{IPCE}(\lambda )=\frac{J_{SC}\times 1240}{I_{\mathrm{light}}(\lambda )\times \lambda }$$

where is the wavelength, I_light_ is the light intensity, the unit of *J_SC_* is mA/cm^2^, the unit of I_light_ is mW/cm^2^ and the unit of *λ* is nm. When the F2-P3CT-Na solution is used, the IPCE values in the green-to-red wavelength range are significantly increased, which can be used to explain the higher *J_SC_* of the resultant solar cells (see Figure 2 and Table 1). Figure 10b shows that the integrated current densities of the P3CT-Na based MAPbI_3_ solar cells are consistent with the averaged *J_SC_* values in Table 1. Figure 11 presents the wavelength-dependent FF and PCE of the P3CT-Na based MAPbI_3_ solar cells fabricated with and without the double-filtering process for the P3CT-Na solution. In the visible-to-near infrared wavelength range, the FF values of the solar cell are higher than 70%, which indicates that the double-filtering process for the P3CT-Na solution can effectively reduce the formation of defects in the bottom region of the MAPbI_3_ thin film. Without the filtering process, the FF values of the solar cell are lower than 60% in the blue-to-yellow wavelength range, which means the formation of deep defects in the MAPbI_3_ thin film because the light absorption in the blue-to-yellow wavelength range is contributed from the electron transitions from the first valence band to the first conduction band and second conduction band [33]. In a MAPbI_3_crystal, the conduction band and valence band are mainly spatially distributed in the Pb cations and I anions, respectively [34]. Without the filtering process, the low FF also results in the relatively low PCE in the blue-to-yellow wavelength range (see Figure 11b). It is noted that a highest PCE of 40.15% is achievedat the wavelength of 650 nm under a low light intensity of 4 mW, which shows the potential for collecting indoor lights [35] due to the low carrier recombination under low light intensities. In other words, the lack of MA cations in the MAPbI_3_crystal can result in the weaker electron transition from the first valence band to the second conduction band, which was confirmed by the Raman scattering spectra from the MAPbI_3_/P3CT-Na interface [36] (see Figure 12). When the F2-P3CT-Na solution is used, the Raman scattering intensities from the PbI modes and MA liberational mode both are increased, which indicates that the crystallinity of MAPbI_3_ in the bottom region can be increased due to the better contact quality at the MAPbI_3_/P3CT-Na interface (see Figure 8).

## 4. Conclusions

In summary, the averaged power conversion efficiency (PCE) of the P3CT-Na based MAPbI_3_ solar cells can be largely increased from 11.72 to 18.92% by improving the contact quality at the MAPbI_3_/P3CT-Na interface with a double-filtering process for the P3CT-Na solution. Compared to the other P3CT-Na based MAPbI_3_ solar cell, the averaged PCE is increased from 16.60 to 18.92% with the use of the double-filtering method. The double-filtering process can be used to reduce the formation of micrometer-sized P3CT-Na aggregates and thereby forming a large-scale and ultra-thin P3CT-Na film on top of the ITO/glass substrate. Besides, the absorbance spectra and photoluminescence data show that the improvement in the *J_SC_* is mainly due to the thicker MAPbI_3_ thin film and more efficient hole collection at the P3CT-Na/MAPbI_3_ interface. It is noted that a high PCE of 40.15% is achieved at the wavelength of 650 nm under a low light intensity of 4 mW/cm^2^, which means that the P3CT-Na based MAPbI_3_ solar cells have the potential to be used in dim-light environment.

## Figures and Tables

**Table 1:** Photovoltaic performance of the P3CT-Na based MAPbI_3_ solar cells with the different filtering processes under one sun illumination (AM 1.5G, 100 mW/cm^2^).

P3CT-Na Solution	V_OC_ (V)	*J_SC_* (mA/cm^2^)	FF (%)	PCE (%)
F0-P3CT-Na	0.961 ± 0.003	19.43 ± 2.32	62.8 ± 5.6	11.72 ± 2.62
F1-P3CT-Na	0.968 ± 0.003	22.16 ± 0.79	68.2 ± 1.8	14.63 ± 0.97
F2-P3CT-Na	1.055 ± 0.003	23.88 ± 0.17	75.1 ± 1.3	18.92 ± 0.52

## Data Availability

The data presented in this study are available on request from the corresponding author.
